# Proposal and Definition of an Intelligent Clinical Decision Support System Applied to the Screening and Early Diagnosis of Breast Cancer

**DOI:** 10.3390/cancers15061711

**Published:** 2023-03-10

**Authors:** Manuel Casal-Guisande, Antía Álvarez-Pazó, Jorge Cerqueiro-Pequeño, José-Benito Bouza-Rodríguez, Gustavo Peláez-Lourido, Alberto Comesaña-Campos

**Affiliations:** 1 Department of Design in Engineering, University of Vigo, 36208 Vigo, Spain; jcerquei@uvigo.es (J.C.-P.); jbouza@uvigo.es (J.-B.B.-R.); gupelaez@uvigo.es (G.P.-L.); acomesana@uvigo.es (A.C.-C.); 2 Design, Expert Systems and Artificial Intelligent Solutions Group (DESAINS), Galicia Sur Health Research Institute (IIS Galicia Sur), SERGAS-UVIGO, 36213 Vigo, Spain; 3 Center for Health Technologies and Information Systems Research—CINTESIS, Faculty of Medicine, University of Porto, 4200-450 Porto, Portugal

**Keywords:** cancer, breast cancer, design, clinical decision support system, intelligent system, expert system, machine learning, decision making, medical algorithm, design science research

## Abstract

**Simple Summary:**

Designing systems that optimize the process of evaluating mammogram images with the goal of improving the diagnostic process of breast cancer is an active field of research due to the large health and social impact of this disease. This paper presents a new intelligent clinical decision support system that, through the concurrence of inferential models, allows the definition of various risk metrics for patients. Those metrics are weighted and combined into a *Global Risk* value to be finally corrected by means of an empirical weighting factor derived from the BI-RADS analysis and condition associated with the patient’s mammogram images. The validation results have shown meaningful disease detection rates within the study group used, which makes it possible to estimate the potential for a diagnostic use of the developed system.

**Abstract:**

Breast cancer is the most frequently diagnosed tumor pathology on a global scale, being the leading cause of mortality in women. In light of this problem, screening programs have been implemented on the population at risk in the form of mammograms, starting in the 20th century. This has considerably reduced the associated deaths, as well as improved the prognosis of the patients who suffer from this disease. In spite of this, the evaluation of mammograms is not without certain variability and depends, to a large extent, on the experience and training of the medical team carrying out the assessment. With the aim of supporting the evaluation process of mammogram images and improving the diagnosis process, this work presents the design, development and proof of concept of a novel intelligent clinical decision support system, grounded on two predictive approaches that work concurrently. The first of them applies a series of expert systems based on fuzzy inferential engines, geared towards the treatment of the characteristics associated with the main findings present in mammograms. This allows the determination of a series of risk indicators, the *Symbolic Risks*, related to the risk of developing breast cancer according to the different findings. The second one implements a classification machine learning algorithm, which using data related to mammography findings as well as general patient information determines another metric, the *Statistical Risk*, also linked to the risk of developing breast cancer. These risk indicators are then combined, resulting in a new indicator, the *Global Risk*. This could then be corrected using a weighting factor according to the BI-RADS category, allocated to each patient by the medical team in charge. Thus, the *Corrected Global Risk* is obtained, which after interpretation can be used to establish the patient’s status as well as generate personalized recommendations. The proof of concept and software implementation of the system were carried out using a data set with 130 patients from a database from the School of Medicine and Public Health of the University of Wisconsin-Madison. The results obtained were encouraging, highlighting the potential use of the application, albeit pending intensive clinical validation in real environments. Moreover, its possible integration in hospital computer systems is expected to improve diagnostic processes as well as patient prognosis.

## 1. Introduction

Breast cancer is currently the most frequently diagnosed tumor pathology worldwide, accounting for one in eight cases of cancer among the overall population. One in four cases of oncologic pathology in women correspond to breast cancer [1,2,3], being the cause of 15.5% of overall cancer deaths by the year 2020 [2]. Due to the high impact of this type of cancer, in the last century, a major effort has been made to implement early detection strategies that, along with early diagnosis as well as effective treatment, have significantly improved the diagnosis of patients and lowered the associated mortality rates [3]. These early detection strategies, usually carried out through population screening programs, are generally based on the performance of periodic mammograms in high-risk groups, mainly women over 40 years of age. The aim is to detect, as soon as possible, any signs that may indicate the possible presence of the pathology, with the subsequent referral of suspected cases for further testing in order to confirm or refute the suspicion and establish a diagnosis. In this regard, for the evaluation of mammograms, the Breast Imaging Reporting and Data System (BI-RADS) [4] is standardly applied, providing the medical team with a common lexicon for the description of the findings observed in the images and facilitating the categorization of the mammogram through the definition of seven levels. These range from no suspiciousness to absolute certainty that the subject has a case of breast cancer, thus facilitating the task of describing and classifying the findings. Nevertheless, the interpretation of mammograms is not trite, and depending on the training and experience of the medical team in charge of evaluation, the results may show a certain degree of variability and subjectivity [5,6,7]. In some cases, this may imply the performance or omission of extra tests to confirm or rule out a potential case of breast cancer, with all the possible disadvantages that this may entail for the patient.

In this context, within the healthcare field, it is essential to have mechanisms and tools available to provide support in the arduous and challenging clinical decision-making processes. These tools, usually supported by artificial intelligence techniques, are currently a working reality, with multiple and diverse proposals existing in the current literature, mostly integrated into clinical decision support systems [8,9,10,11,12,13,14,15,16,17,18,19,20,21,22,23,24,25]. With regard to breast cancer, many proposals make use of symbolic inference models [21,25], although with the rise of the connectionist paradigm, in recent years applications have been developed that use large and complex neural network models for the detection of these tumors [26,27,28,29,30,31,32]. They have enabled the management of hospital resources to be improved upon, thereby increasing the quality of the services provided while reducing the high costs associated with them. Along these lines, this paper proposes an adaptation, development and proof of concept of the work presented by the authors in Casal-Guisande et al. (2022) [25]. In that publication, the authors outlined the concept of a breast cancer diagnostic system based on the implementation of a cascade of expert systems that collected and formalized the patient’s medical information. Their output was processed to find covariance factors that would form a new knowledge base to train a statistical classifier upon which the final diagnostic prediction could be based. The sequential flow of information that was proposed reduced the uncertainty related to both processing and interaction, although it diminished the inferential counter position. In other words, the statistical inference was set off after the symbolic inferential process, whose outputs were the grounds for the definition of the classifier’s starting data. Therefore, there is a conditioning of the symbolic part to the statistical part that, in a sense, limits the diversification of knowledge and, above all, limits the interpretation of the correlation patterns that the machine learning algorithm can obtain.

The improved system proposed in this paper is built upon the aforementioned one and aims not only to improve upon the results published at the time, but also redesign the flow of data and knowledge in order to increase the diversifying effect of the inferential models. To this end, an intelligent system was implemented consisting of two blocks that work concurrently [12,13,17,25,26,27]. The first block is based on the use of expert systems employing fuzzy inference engines, focused on the processing of data related to the findings present in mammograms, i.e., masses, calcifications, asymmetries and distortions of the architecture. The second block, based on the use of a classification machine learning algorithm, is focused on the joint processing of data from mammograms, with the exception of BI-RADS, as well as general patient data. Through these concurrent processes it is able to obtain a series of risk indicators, the *Symbolic Risks* and the *Statistical Risk*, respectively. These indicators are then aggregated to obtain a percentage indicator of *Global Risk*, representative of the likelihood of developing breast cancer. This might then be corrected and its value weighted according to the BI-RADS level assigned by the medical team, thus determining a *Corrected Global Risk* indicator that, after adequate interpretation and evaluation, allows for the generation of warnings regarding the patient’s condition as well as provides recommendations. The simultaneous deployment of the inferential systems enables their outputs to be contrasted and merged, while the development of analytical models, as will be discussed later, can effectively reduce the uncertainty associated with this type of structure.

This document is organized in five sections. Section 2 presents the conceptual design of the proposed intelligent system, outlining the different stages and data flows. It is then followed by a thorough description of the software implementation of the intelligent system in addition to commenting on the previous results of its proof of concept. In Section 3, a practical case of application of the proposed intelligent system is presented so as to illustrate its performance. Section 4 discusses the architecture of the proposed system, followed by Section 5, which presents the conclusions obtained along with potential development lines.

## 2. Materials and Methods

### 2.1. Definition of the System

#### 2.1.1. Database Description

In order to conduct this research, a database belonging to the School of Medicine and Public Health of the University of Wisconsin-Madison was used containing information on 130 patients. Out of the 130 patients within the dataset, 21 were diagnosed with breast cancer after comprehensive testing. All patients in this study underwent confirmatory biopsies that, in each case, either validated or ruled out the presence of cancer from a histopathological point of view. This dataset was comprised of general patient information (age, family and personal history of cancer), commonly available on electronic health records, as well as mammography profiling via BI-RADS terminology [28]. It also included the associated BI-RADS category.

A summarized overview of the main characteristics of the population used can be found in Table 1. A further detailed description of the data considered is presented in Table 2 and Table 3.

#### 2.1.2. Conceptual System Design

**Figure 1:**
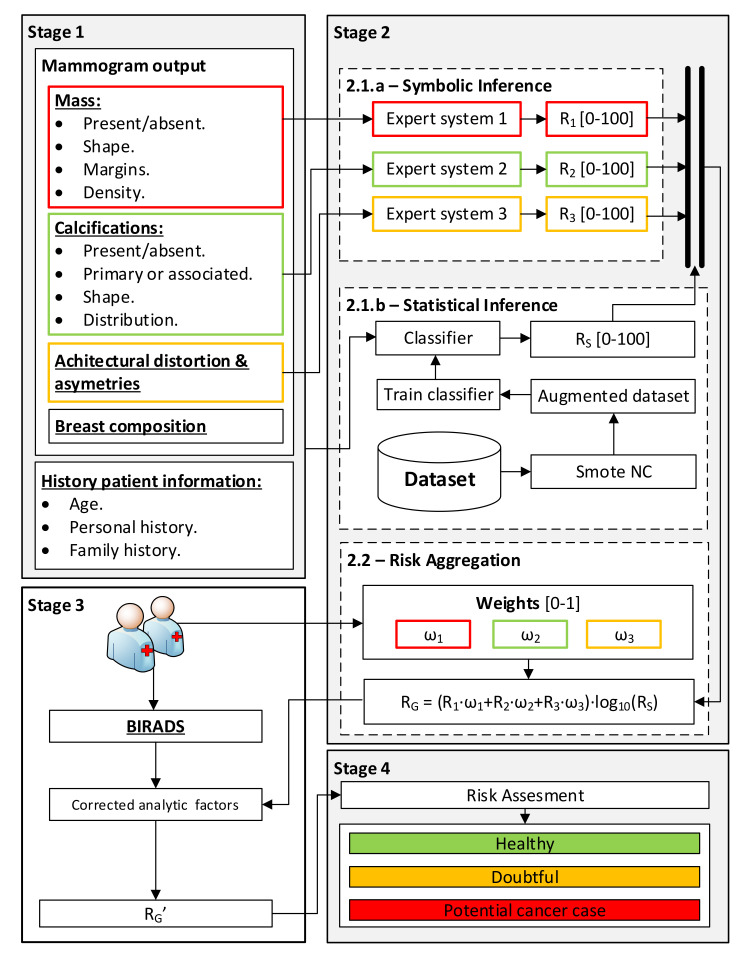
Flowchart for the intelligent clinical decision support system for the determination of breast cancer risk. The flow of information that takes place in the different stages contemplated by the system can be observed, including Stage 1, data collection, Stage 2, subdivided into Stage 2.1.a, symbolic inference, Stage 2.1.b, statistical inference, and Stage 2.2, risk aggregation. Following this, Stage 3 carries out the determination of the *Corrected Global Risk* based on the value presented by the BI-RADS indicator. Lastly, Stage 4 is where warnings are issued and decisions are taken.

A flowchart for the intelligent clinical decision support system for breast cancer risk evaluation is shown in Figure 1.

##### Gathering and Interpretation of Patient Information

The first stage in the proposed intelligent system is restricted to the collection of general patient data, typically available in electronic health records (for more information see Table 2) as well as the interpretation and annotation of the findings found in mammograms following the BI-RADS^©^ terminology [28] by a medical specialist, detailed in Table 3. Together with each of the descriptors, a corresponding field associated with the nature of the data is added, differentiating between numerical and categorical data.

##### Data Processing

Once the data presented in Stage 1 have been compiled and arranged, they are then processed by the proposed intelligent system, firstly employing a series of expert systems and a classification machine learning algorithm arranged in two sub-stages that work concurrently [12,13,17,25,26,27], 2.1.a and 2.1.b. Through these two sub-steps, it is possible to determine a series of risk indicators related to the risk of developing breast cancer. These same indicators are then aggregated in Step 2.2, which determines a *Global Risk* index that combines them and represents the risk of a patient suffering from breast cancer. The different sub-stages are detailed below:

- Stage 2.1.a—Determination of *Symbolic Risks*: once the information regarding the findings in the mammogram, introduced in Stage 1, has been gathered, the processing is carried out using a series of expert systems that work concurrently [12,13,17,25,26,27] which are based on Mamdani-type fuzzy inference systems [29,30,31]. Each of these expert systems is assigned the processing of the data subsets associated with the different findings (masses, calcifications, asymmetries and distortions) in order to obtain a series of risk indicators, the *Symbolic Risks* (*R*_1_, *R*_2_ and *R*_3_), with values ranging between 0 and 100, each of them related to the risk of developing breast cancer.
- Stage 2.1.b—*Statistical Risk* determination: In parallel to Stage 2.1.a, Stage 2.1.b carries out the processing of all the collected data, both those in Table 2 and Table 3, excluding the BI-RADS category determined by the expert, by means of a machine learning classification algorithm [32]. According to the nature and quality of these data, they may be subjected to a normalization process with a possible synthetic scaling of the sample [25]. The algorithm training is based on the dataset introduced in Section 2.1.1, where each case is labeled as either *“cancer”* or “*non-cancer*”. This allocation is indisputable within the study group since all the patients underwent a biopsy and their real condition is known. This considerably reduces the epistemological and interaction uncertainty of the training data itself. Once the model has been trained, a new patient’s data are presented, so that the model may determine a percentage indicator of risk at the output, the so-called *Statistical Risk* (*R_s_*), ranging from 0 to 100, which is intended to indicate the risk that the patient may suffer from breast cancer.
- Stage 2.2—Risk aggregation and *Global Risk* determination: Having obtained the *Symbolic Risks* (*R*_1_, *R*_2_ and *R*_3_) as well as the *Statistical Risk* (*R_s_*), they are then aggregated by means of the expression shown in Equation (1), which allows for the calculation of the *Global Risk* (*R_G_*). Said expression is based on the product of the weighted sum of the *Symbolic Risks* and the decimal logarithm of the *Statistical Risk*. The first term, the weighted sum, provides a measure of risk that brings together the different *Symbolic Risk* indicators according to the potential importance given by the medical team to each of the groups of findings (masses, calcifications and asymmetries, and distortions of the architecture). Meanwhile, the second term has a multiplicative effect, increasing the level of risk previously obtained in the event that the patient under analysis presents a similar pattern to that of a patient with breast cancer within the sample with which the statistical model was constructed. Note that in the event that any of the groups of findings used to calculate the *Symbolic Risks* is absent in the case under study, meaning that any of the risk indicators is null, an equitable weight redistribution will be performed among the rest of the findings. Alternatively, a new weight redistribution proposed by the medical team will be considered. Furthermore, it is also worth mentioning that the *Global Risk* value ranges between zero and one hundred; in case of a higher value, despite the multiplicative effect of the logarithm term, its maximum value shall be one hundred.

(1) $$R_{G}=\left(\omega_{1}\cdot R_{1}+\omega_{2}\cdot R_{2}+\omega_{3}\cdot R_{3}\right)\cdot \log_{10}R_{s}$$

##### Global Risk Correction

As discussed in the previous stage, a series of risks were calculated and aggregated, resulting in the *Global Risk* being determined. Following this, during this stage, the *Global Risk* could be corrected by taking into account the value of the BI-RADS index proposed by the medical team according to the analysis of the mammograms. This *Global Risk*, as has been stated, is a measure of the patient’s potential risk of developing breast cancer, computed by means of statistical categorical data as well as assorted symbolic variables related to findings from the mammograms. While it is an effective metric, it is preferable to use the internationally validated BI-RADS indicator, previously presented. Currently, this indicator is the standard for tumor assessment in breast cancer, being the main diagnostic criterion. Nevertheless, the interpretation of the index itself, and the allocation of an index to a potential tumor lesion, requires considerable expertise and accuracy on the part of the medical team. Both knowledge and experience in the use of the BI-RADS scale are required, which makes its widespread use difficult in medical teams with little practice in its use or in the diagnostic interpretation. Still, as has been stated, its use continues to be crucial in the staging of breast cancer and should therefore be valued and accepted as a fundamental diagnostic vector. This study, in not including the BI-RADS index when calculating the *Global Risk,* aims towards the formalization of knowledge, both in its statistical and symbolic aspects, to be precise. Thus, the diagnostic process would not depend on the determination of highly specific knowledge, but rather on a more general formalization of the data relating to the patient’s health. This would allow a plausible and reliable risk level to be obtained, with less epistemic uncertainty associated with the application of the BI-RADS index. However, the influence of the BI-RADS cannot be ignored under any circumstances; hence, it is proposed that once the *Global Risk* has been determined, it should be weighted in an orderly manner by the BI-RADS index that the medical team associates with the possible cancerous tumor that the patient may suffer from. To achieve this, a weighted order algorithm was adapted in which, depending on the BI-RADS index obtained, factors that rescale the *Global Risk* are established according to the risk itself. This allows us, on the one hand, to limit the uncertainty of the index and, on the other, to reaffirm the implicit closeness that should exist between the *Global Risk* and the BI-RADS index. Equation (2) presents the expression for the calculation of the *Corrected Global Risk* (*R_G_’*), where $F_{p}$ is the weighting factor.

(2) $$R_{G}’=R_{G}\cdot F_{p}$$

Table 4 presents a proposal of the *Global Risk* weighting factors according to the order of the BI-RADS index assigned to the patient. The determination of the factors was derived from an empirical analysis of the correlation and causality chains existing between the presence of cancer, the BI-RADS index and the *Global Risk* value determined. The aim was to obtain an analytical relationship that would allow the *Global Risk* value to be modified as a function of the BI-RADS index, thereby maximizing its diagnostic accuracy. With this, the aim is not only to improve the accuracy of the prediction, but also to bring said prediction closer to the standard medical interpretation derived from the BI-RADS index value, often determinant in the confirmation of a suspected disease case. Precisely due to this, the weighting factor does not contemplate any statistical inferential process, since it exclusively involves an analytical multiplication or division factor associated with what the BI-RADS index means and represents. It is important to note that despite the application of this weighting, the absolute maximum value of the *Corrected Global Risk* equals one hundred.

###### Generation of Warnings and Decision Making

Once the *Corrected Global Risk* (*R_G_’*) is determined, its evaluation is passed on, thereby setting up a series of statuses and recommendations related to each patient:

- Healthy case: Refer the patient for routine review;
- Dubious case: Reconsider the patient’s case, consider performing other tests as well as summoning the patient for a new visit in a period of time to be specified;
- Potential breast cancer case: Perform confirmatory tests.

The evaluation is a simple suggestion of classification that obeys the need to make an explicit diagnostic decision. Although it is supported by the *Corrected Global Risk*, it could equally be supported by the *Global Risk* as long as the medical team does not consider the correction necessary.

### 2.2. Implementation of the System

This section deals with the implementation of the intelligent clinical decision support system for breast cancer diagnosis, describing the proposed software application in detail and commenting on the previous results derived from its proof of concept. The system was developed following the recommendations of Hevner et al. [33,34], which would allow for integration into hospital information systems, if needed.

MATLAB© software (R2021b, MathWorks©, Natick, MA, USA) was used to carry out the implementation. Table 5 shows a list of the different Toolboxes used.

In addition to these tools, it was necessary to use Python (version 3.9.12) as an auxiliary tool to provide support for the data augmentation process by means of the SMOTE-NC algorithm from the imbalanced-learn library [38].

**Figure 2:**
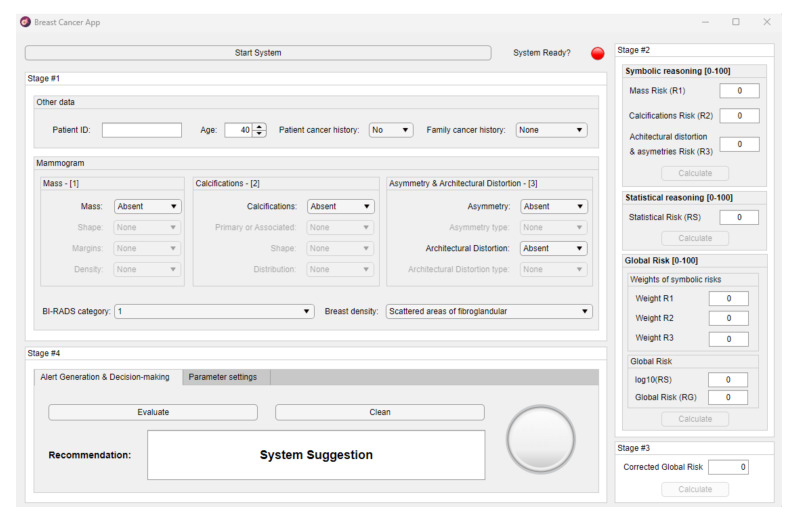
Screenshot of the main graphical interface of the intelligent clinical decision support system for breast cancer risk assessment. Box *Stage #1* refers to the first stage of the methodology in which data collection is performed. Box *Stage #2* refers to the second stage of the methodology in which the system risk assessment is performed. In the *Stage #3* box, the *Global Risk* indicator is corrected by considering the value of the BI-RADS indicator. Finally, *Stage #4* refers to Stage 4, in which the generation of alerts and decision making is carried out.

Figure 2 depicts a screenshot of the home screen of the developed software application. The *Stage #1* box corresponds to the area of the application used to enter the starting data, i.e., the patient’s general data and those related to the findings coming from the mammography interpretation by the specialists. The *Stage #2* box corresponds to the area of the application used to calculate the risks. This represents Stage 2 of the methodology presented, with three different boxes: the Symbolic Reasoning box, relating to Stage 2.1.a, in which the *Symbolic Risks* associated with the groups of findings present in the mammograms are determined, the Statistical Reasoning box relating to Stage 2.1.b, associated with the calculation of the *Statistical Risk*, and finally the *Global Risk* box, relating to Stage 2.2, in which the aggregation of the *Symbolic Risks* and *Statistical Risk* is carried out, yielding the *Global Risk*. Then, in the *Stage #3* box, the *Global Risk* is corrected, taking into account the value of the BI-RADS indicator. Finally, the *Stage #4* box presents the panel related to Stage 4 of the intelligent system, which deals with the generation of warnings and decision making.

#### 2.2.1. Data Collection

Once the data of each of the patients being studied by the system are submitted, they must be input into the software through the available fields in the *Stage #1* box in Figure 2. There are two areas: one relating to general patient information in the “Other data” section, and the other to information regarding the findings observed in the mammograms, as well as the BI-RADS indicator established by the medical team after the evaluation of each case, which must be entered in the “Mammogram” section. This information should be typed into the forms with caution, avoiding errors or omissions that could lead to an inflated printout, thus increasing the uncertainty of the system.

#### 2.2.2. Data Processing

After inputting the data into the application, they are processed by the proposed intelligent system. In the application there is a panel, the so-called *Stage #2*, where the different calculation blocks are located. In line with what was discussed in Section 2.1.2, this processing consists of two main blocks that work concurrently [12,13,17,25,26,27]. The first has a series of expert systems that also work concurrently [12,13,17,25,26,27], focused on the determination of the *Symbolic Risks*, while the second deploys a classification machine learning algorithm, focused on the determination of the *Statistical Risk*. Following this, both the *Symbolic Risks* and the *Statistical Risk* are aggregated, determining the *Global Risk*.

Next, the definition associated with each of the blocks is described, as well as the calculation of the previously mentioned risk indicators.

##### Determination of the Symbolic Risks

As mentioned before, a number of concurrent expert systems are used to determine the *Symbolic Risks* [12,13,17,25,26,27]. These systems are in charge of processing the different groups of data relating to the findings present in mammograms (masses, calcifications and asymmetries, and distortion of the architecture), already presented in Table 3.

In this paper, Mamdani-type fuzzy inference engines [29,30,31,39] are employed, similar to those used in the first level of the cascade in the work of Casal-Guisande et al. [25] and others [12,13,17,26,27].

Table 6 summarizes the antecedents and consequents of each of the deployed expert systems. Regarding the membership functions, we complied with the recommendations of Ross [39], choosing the use of normal, convex and symmetric functions. As for the antecedents, singleton membership functions were used, while in the case of the consequents, those related to *Symbolic Risks*, triangular membership functions between 0 and 100 were used. The choice to use singleton functions for the antecedents was due to the nature of the data represented, as they point to a single value within a category, such as the shape of a mass within the typified ones. On the contrary, the consequents are represented by triangular functions since their variables can take membership values between zero and one, but there is only a single value that presents maximum membership. The risks, intended here as the consequent variables, were defined by assuming that there is a single value within their measurement scale that represents the highest risk, in line with the latest work published by the authors in this field [25].

A general summary of the overall configuration of the expert systems’ inference engine is presented in Table 7.

Once the symbolic inference process is carried out, three risk indicators are obtained, *R*_1_, *R*_2_ and *R*_3_, each one of them related to the risk of developing breast cancer according to the group of findings associated with their respective calculation. The greater the value of each of them, the greater the risk of developing breast cancer.

##### Determination of the Statistical Risk

Concurrently [12,13,17,25,26,27] to the determination of the *Symbolic Risks*, the determination of the *Statistical Risk* is carried out through the use of a classification machine learning algorithm. This model is based on the dataset presented in Section 2.1.1, with the exception of the BI-RADS indicator established by the medical team. Most of the data are categorical [40,41], as can be seen in Table 2 and Table 3. The only exception is age, which is normalized using the Min-Max normalization method, the expression of which is shown in Equation (3).

(3) $$t’=\frac{t_{i}-\min\left(t\right)}{\max\left(t\right)-\min\left(t\right)}$$

Upon revision of the data set distribution, a significant imbalance between the “*cancer*” and “*non-cancer*” classes is apparent, which may affect the performance of the classifier and its subsequent generalization. In light of these circumstances, and following the usual trend in the development of decision support tools for medical diagnostic environments, it was decided to use controlled data augmentation techniques, through which the results of the binary classifiers are improved [25,42]. In this paper, the use of SMOTE-NC, a variant of the synthetic minority over-sampling technique (SMOTE) [42,43], is employed. This approach allows for the augmentation of datasets in which numerical and categorical variables exist simultaneously once all of them have been transformed [25]. The data augmentation strategy adopted involves adding patients until there were 200 cases of each class (*cancer* and *non-cancer*) using a strategy with a number of neighbors k = 5 [25].

**Figure 3:**
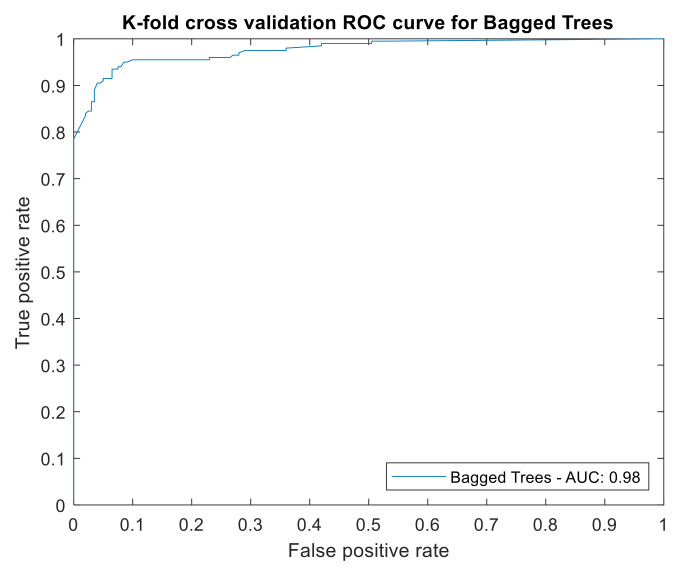
ROC validation curve for the cancer class of the bagged trees model.

By doing so, a coherent and representative dataset was assembled to be used for the training and tuning of the classification machine learning algorithms. Several tests were performed using the MATLAB^©^ Classification Learner app, with a k-fold cross validation strategy [44], with k = 5. After carrying out different tests, and relying on the use of ROC curves [25], it was found that the bagged tree algorithm was the one that demonstrated the best results. Regardless, it is worth noting that any other machine learning classification approach might be a valid alternative as long as it provides better results, in terms of the interpretation of the ROC curves, than those obtained with the current approach. Figure 3 shows the ROC validation curve of the bagged trees algorithm for the “*cancer*” class, showing a very high AUC [25] value of 0.98.

After defining the model, the classifier returns a risk indicator, the so-called *Statistical Risk*, corresponding to the risk of a new patient developing breast cancer. It ranges from zero to one, although for the sake of convenience it is scaled between zero and one hundred. The greater the *Statistical Risk* value, the greater the risk that the patient is suffering from breast cancer.

##### Determination of the Global Risk

Having determined the *Symbolic Risk* as well as the *Statistical Risk*, their aggregation is carried out using Equation (1), as presented in the conceptual description of the system. This results in a new risk indicator that groups together the previous risks, known as *Global Risk*. Its value lies between zero and one hundred, representing, similarly to previous cases, the risk of suffering breast cancer. However, in this instance, the indicator is aggregated, bringing together the different findings and general data of the patient under different perspectives, both symbolic and statistical.

##### Determination of the Corrected Global Risk

As mentioned earlier, in this study, the BI-RADS indicator was not included in the calculation of the risk indicators. Despite this, it is not reasonable to ignore its influence, so once the *Global Risk* is determined, it is weighted according to the BI-RADS index allocated to the case by the medical team. The objective of this correction does not lie in improving the real predictive capacity of the system, an issue that is implicitly covered in the determination of the *Global Risk*, but in bringing the usual medical decision process based on the BI-RADS standard closer to the decision itself. Since it is common practice to refer patients based mainly on the values of this index, the system adopts this influence and generates a corrected risk value that is close to said practice, even though it might differ from the real prediction provided by the system. In this sense, a series of empirical expressions were defined that make it possible to correct the predictions to bring them closer to the suspicion signs inherent to BI-RADS. Table 4 shows the correlation between the BI-RADS level and the expression used to determine the *Corrected Global Risk* indicator.

#### 2.2.3. Generation of Warnings and Decision Making

Once the patient’s data are processed and the *Global Risk* indicator is determined and corrected in accordance with the BI-RADS level assigned to the case, an assessment of the existing risk is carried out with the aim to establish the patient’s status in order to provide recommendations that will facilitate the patient’s diagnosis. In this sense, three possible states are considered, which are summarized in Table 8, together with the established risk thresholds. It should be underlined that these risk criteria may be subject to revision in the future depending on the results of the clinical validation stage, as well as on the criteria of the medical specialists.

It is worth to emphasize that the risk levels established here are just illustrative and obey only the need to carry out an explicit diagnosis and the potential application of the *Global Risk* correction. As already mentioned, the system has classification capabilities represented by the *Global Risk* that, in principle, would not require risk thresholds, since it classifies patients into two classes: those who could suffer from cancer and those who could not. However, by incorporating a correction based on the BI-RADS index, this classification is vaguer to approach and adapt to the criteria of the diagnostic medical team. For this reason, it is considered convenient to keep a suggestion of risk levels, which is much closer to the interpretation of the doctor than to the inferential classification of the system.

#### 2.2.4. Analysis of Results

After the implementation of the system and taking as a reference the collected and conveniently labeled dataset, a proof of concept [45] was carried out to demonstrate the correct operation of the software prototype developed, as well as estimate its capabilities and diagnostic success. The system can be understood as a binary classifier [46,47] that predicts the assignment of classes, two in this case: “*cancer*” or “*non-cancer*”, to the patients included in the study. Likewise, this prediction must be reflected, first in the calculation of the *Global Risk*, and second in the determination of the *Corrected Global Risk*, since in reality both risks start from different premises. As already mentioned, the former is determined from the patient’s data through an inferential process, while the latter consists of an analytical approximation to the usual medical practice based on the BI-RADS index. To measure the efficiency and performance of this classifier [46,47] the standard measures of sensitivity (a metric for the ability to detect the disease in patients actually suffering from the disease) and specificity (a metric for the ability to not detect the disease in patients actually not suffering from the disease), were used. Furthermore, a global precision metric was added that measures the general performance of the classifier, in this case through the Matthews correlation coefficient (*Mcc*) [48,49,50]. On the other hand, additional and complementary metrics to the usual ones were incorporated, such as the false negative rate (a metric for those cases incorrectly classified as not suffering from the disease), and the false positive rate (a metric for those cases that the classifier incorrectly identifies as suffering from the disease). Other values, such as the sensitivity per lesion (a metric for the fraction of correctly identified tumors) were, in this case, integrated into the metrics described above. All the previous values are collected in Table 9.

From the analysis of the results shown in that table, it is possible to draw different conclusions. Analyzing the values obtained in the calculation of the *Global Risk* on the study dataset, high values of sensitivity (90.5%) and specificity (89.81%) are observed, which also results in high values of the Matthews correlation coefficient (0.7). Taking these data, it is reasonable to conclude that the system presents, in this proof of concept, unique and differentiating predictive capabilities supported by the concurrence of inferences. The ability to model knowledge through symbolic models, while incorporating the results of statistical inference as well, undoubtedly increases its performance as a classifier.

On the other hand, considering the metrics derived from the *Corrected Global Risk*, sensitivity values of 100% and specificity of 60.19% are observed with a Matthews correlation coefficient value of 0.44. It is clear that the predictive capabilities of the classifier decreased. However, in the same way, its cause is also evident: as mentioned before, the correction was not intended to improve the results over the real prediction, but to bring them closer to the usual medical and diagnostic practice. In other words, the goal is that the system can behave in a standardized and recognizable way for the healthcare team that uses it. Thus, the classification strongly obeys the BI-RADS index and, based on it, supports subsequent decisions, which would guarantee 100% success in the detection of the disease, even assuming an increase in the number of patients undergoing unnecessary confirmatory tests. The data obtained give a clear reflection of what has been said.

Therefore, the intelligent system has two characteristics that are differentiated and very useful in diagnosis. On the one hand, it has enhanced predictive capabilities with excellent results on the study dataset. On the other hand, the system can adapt these results to the usual behavior of medical teams in the diagnosis of breast cancer and maximize the detection of all suspected cases. The potential of use and diagnosis of the system are thus highlighted and verified in the proof of concept, which thus fulfills the main objective of its realization.

Despite all this, it should be noted that those are only preliminary results derived from the proof-of-concept analysis on the study dataset. It is foreseeable, and reasonable, that the incorporation of new data, unrelated to the reliability and low uncertainty of the data used, decreases the predictive capacity of the system, although the correction strategy can always guarantee detection.

## 3. Results

In this section, the intent is not to validate the proposed intelligent system, but rather to present a practical case study of its performance to illustrate its potential use in the clinical setting as well as its ease of use. As commented in the previous section, the scope of our work only spans the proof-of-concept stage, so this example, derived from it, just aims to show the application workflow using data from a new patient. Thus, it is of note that the patient’s data analyzed in this scenario were not involved in the statistical model training process.

### 3.1. Data Collection

A summary of the findings in the mammography, as well as the general data of the patient, are presented in Table 10. With the intention of comparing the results of the model with the real clinical picture presented by this patient, it is essential to bear in mind that this was a patient who, after diagnostic tests, was found to have breast cancer.

Having fed the data into the app, it was then processed by the intelligent system.

### 3.2. Data Processing

Once the data were submitted to the application, they were processed by the proposed intelligent decision support system. Firstly, the *Symbolic Risk* indicators and *Statistical Risk* indicator were determined. As for the *Symbolic Risks*, values of 89.97%, 99.98% and 0% were obtained for *R_1_*, *R_2_* and *R_3_*, respectively. As for *Statistical Risk*, it had a value of 25.61%.

These risks were then aggregated by means of Equation (1). It was assumed that all the *Symbolic Risks* were equally influential. However, since the third risk had a null value, the weights associated with each *Symbolic Risk* were as follows: $\omega_{1}=\omega_{2}=0.5$, $\omega_{3}=0$. Equation (4) shows the numerical calculation of the *Global Risk*, which in this case, because of its upper bounded value, presented a value of 100.

(4) $$R_{G}=\left(0.5\cdot 89.97+0.5\cdot 99.98+0\right)\cdot \log_{10}25.61\;\geq 100\to \;R_{G}=100$$

**Figure 4:**
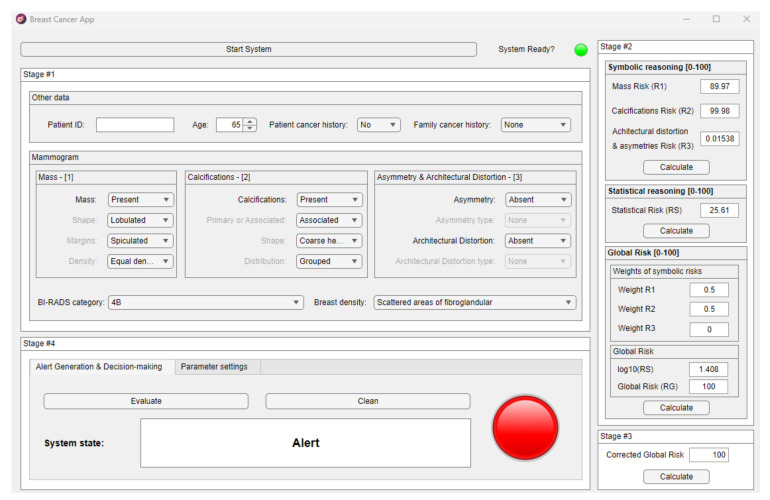
Screenshot of the software tool for the case study.

A screenshot of the *Stage #2* panel in the application is shown in Figure 4, in which the obtained risk values are displayed.

### 3.3. Global Risk Correction

Having obtained the *Global Risk*, in *Stage #3*, it was corrected by applying a weighting according to the BI-RADS level assigned by a specialist. Since the BI-RADS level assigned was 4B and the *Global Risk* already was the maximum value, the *Corrected Global Risk* value was identical, with a value of 100. The obtained *Corrected Global Risk* value can be seen in the *Stage #3* box in Figure 4.

### 3.4. Warning Generation and Decision Making

Lastly, the risk assessment was carried out. A value higher than the second threshold was found, generating a status of maximum alert, as can be seen in the *Stage #4* box in Figure 4. A recommendation was made to the medical team to carry out tests to verify the potential diagnosis. As this example shows, the correction confirmed the suspicion derived from the inferential process that, without any doubt, placed the patient at high risk of suffering from breast cancer. In this case, the correction based on BI-RADS could even be considered unnecessary, but it was carried out in order to consider and adapt the system to the team’s standard diagnostic criteria.

The system’s recommendation was consistent with the verified patient’s condition.

## 4. Discussion

Breast cancer is currently the world’s major diagnosed tumor disease, overtaking lung cancer to be one of the leading causes of death among women. The early detection of this pathology is crucial in reducing its associated impact by means of population screening programs. These consist of mammography screening of high-risk groups in order to detect potential cases and treat them as quickly as possible if needed, thus improving diagnoses and reducing the mortality rates associated with the disease. When interpreting and assessing mammograms, it is standard practice to use the BI-RADS system, establishing a common lexicon as well as a series of levels for categorizing the patient’s lesion, in an attempt to represent the likelihood that a malignant case of cancer is present. Despite this, the interpretation and evaluation process of mammography findings involves a certain degree of variability associated with the experience and training of the medical team in charge of their interpretation. This directly influences the diagnosis and the next steps to be taken regarding each patient.

In this paper, a new intelligent system for clinical decision support in breast cancer is presented as a result of the evolution of the architecture of the intelligent system proposed by the authors in Casal-Guisande et al. [25]. The aim is to adapt it to the particular needs of medical teams in order to facilitate its future validation and improve its performance. The knowledge bases were adapted to the medical team, optimizing the information flows and customizing the system to their particular needs.

Breast cancer diagnosis is a multi-variable problem, in which a series of variables are used to determine whether or not a patient is suffering from the condition. The conventional approach usually employed in these cases, which is the most common in terms of state-of-the-art knowledge, relies on the use of single inferential approaches, either statistical or symbolic. In line with the latest works by the authors [17,25,27], it has become increasingly common and convenient to jointly use inferential approaches of a heterogeneous nature, both statistical and symbolic. In this case, this is carried out through the joint use of a series of fuzzy inferential engines and a machine learning algorithm for classification, representing the diagnostic procedure of breast cancer from different but complementary approaches that seek to represent the same reality. However, from the point of view of the architecture of the proposed intelligent system, and in contrast to the previous work proposed by the authors in Casal-Guisande et al. [25], the use of the symbolic and statistical inferential engines is not sequential, but concurrent. This allows the simultaneous determination of a series of risk indicators associated with the different blocks, the *Symbolic Risks* and the *Statistical Risk*, which are associated with the risk of suffering from breast cancer.

This new approach uses the knowledge needed for calculation through two parallel and concurrent inferential processes, which represents a considerable departure from the use of such knowledge into a single inferential process, the outputs of which could feed further processes. An individual analysis of the different risks could allow the medical team to gauge the risk of a patient developing cancer. However, the individual inferential capability of the different engines deployed is the only one that would be given preference. Nevertheless, in this paper, thanks to the concurrence of the inferential engines, an approach that gives priority to the joint use of the engines can be opted for by using an empirical expression, namely Equation (1), to facilitate the joint interpretation of the engines through a risk indicator, the *Global Risk*. This indicator is based on the weighted sum of the *Symbolic Risks*, each one representing the main groups of findings present in the mammography (masses and calcifications, as well as asymmetries and distortion of the architecture), which allows a medical team to fine-tune the significance and contribution of each of the findings groups towards the aggregated risk, represented by the *Global Risk* indicator. The ability to make this adjustment is a remarkable feature, since it allows customizing and tailoring the use of the system to suit each medical team, making it more versatile.

Moreover, *Statistical Risk* has a multiplicative effect, increasing the existing risk level (obtained through the weighted sum of the *Symbolic Risks*), thereby increasing the hazard level in suspicious cases. With this approach, the different types of risk can be aggregated in a simple and efficient way, incorporating an additional layer to the risk layer associated with the reasoning, thus increasing the risk level in the case that a pattern similar to that of the cancer cases used to train the classification machine learning algorithm is detected.

Undoubtedly, one of the greatest advantages of these inferential models is their almost unlimited capabilities to incorporate knowledge into their knowledge base, expressly formalized or simply in the form of a dataset. This knowledge can always be expanded and used in the prediction once it has been properly analyzed.

Notably, the BI-RADS level assigned in this study by the medical team to each patient does not have a direct influence on the inferential processes. In contrast to the previous work proposed by the authors, it is not an input to the inferential processes. In this case, this assigned BI-RADS level allows weighting the *Global Risk* level obtained by means of the use of an analytical function, thus making possible to reduce or increase the risk level obtained in accordance with the opinion of the medical team.

Hence, the proposed approach is not only a reframing of the already published breast cancer diagnostic process, but an entirely novel conceptualization of it. The shift from a sequential flow of knowledge to a concurring flow is a remarkable breakthrough. Whereas the use of a sequence of inference processes guarantees a preservation of information, adopting a concurrent sequence of inference, fostered by the same knowledge, provides a notable diversifying effect in addition to the aforementioned preservation of information. Regardless of the inferential model used, its prediction results should be unique when it aims at a diagnosis given the same initial input. Fluctuation will always imply an unbounded increase in the uncertainty of the process. Whereas in the sequential approach this could be corrected by factorial techniques, a concurrent approach requires interpretation of the outputs obtained from the inferential processes.

Said interpretation, which is not free of variability, should be carried out, at least initially, through analytical models that adjust the correspondence between their actual values and the predictions. Indeed, the presence of analytical solutions reduces the influence of the generalist artificial intelligence approach by simplifying, if not linearizing, a deductive process. Yet, in this case, the aim is not to find an analytical expression for the prediction, but rather to look for one that will allow the predictions to be combined. Subsequent empirical analysis aims to limit the uncertainty of variability by using simple mathematical relationships, which may seem somewhat perplexing. The use of probabilistic models, Bayesian classifiers, or management of this uncertainty through fuzzy logics, while effective in slightly improving the robustness of the expressions, would not represent a substantial change, as it would be impossible to find realistic relationships between the degrees of certainty of the explanatory variables, in this case the risks, and the explained variable, here the presence or absence of cancer.

Perhaps once a significant number of triads of variables and labels are gathered, annotated and classified, it might be possible to carry out multivariate analyses and find implied causal relationships. However, in a concept proposal such as the one presented in this paper, this question is hardly feasible. Without doubt, this is a novelty, although it is also a weakness (perhaps the most representative of all) of the current formulation.

All the circumstances discussed so far constitute a clear departure from the previous work proposed by the authors, while also representing a first in this field of study.

In addition to all the above, the conceptualization of the proposed intelligent system, supported by the use of symbolic inferential approaches (through the use of expert systems) and statistical inferential approaches (through the deployment of a classification machine learning algorithm), allows for the improvement and optimization of decision-making processes. In this case, this knowledge usually resides in specialized medical teams, so this app facilitates achieving a common diagnostic process by medical teams with different training and experience. Likewise, the intelligent system carries out an implicit control and management of the uncertainty present in the diagnostic process, both in its epistemological and random aspects, as well as considers ambiguity and interaction [51,52]. It does so through the joint use of the aforementioned symbolic and statistical approaches, a trend that is becoming pervasive in the field of intelligent systems.

Besides all the advantages and matters discussed so far, the use of an intelligent system such as the one proposed in this work presents important improvements in the diagnostic and healthcare fields. Determining a percentile risk indicator for breast cancer is a very valuable metric, helping the medical team with the arduous task associated with the early diagnosis of a possible case of breast cancer. This in itself reduces, as far as possible, the false positive and false negative rates during the screening stage, decreasing the associated costs and improving upon the quality of the services rendered. However, although this reduction in false classifications is transcendental in order to avoid unnecessary tests to patients, with the consequent relief not only in their state of mind but also in costs and medical processes, the incorporation of the *Global Risk* correction endows the intelligent system with a unique empirical approach. This means that, although having a diagnostic method with high precision and success is the main motivation of intelligent clinical decision support systems, by incorporating analytical and empirical approaches into their architecture, we succeed in bringing their capabilities closer to the usual medical practice. With this, and whenever the healthcare team considers it necessary, they can maximize detection, sacrificing diagnostic accuracy even in those cases where the system clearly classifies a patient as healthy but their BI-RADS index raises some suspicion. The corrective approach does not improve the classifier, but increases its trust on the part of the team, who can see their own criteria reflected in the system’s predictions, even when these, for example, are excessively conservative. The system could, allegedly, produce minor discrepancies, especially in doubtful cases, based on this correction, although it would always allow the medical team to observe the real prediction, compare, learn and reason based on it.

On the other hand, for the proposed system, in addition to the difficulties previously expressed and related to the reduction of diversification associated with the use of the empirical corrective approach, there are other limitations that must be commented. Obviously, the first one is associated with the formalization of knowledge and the generation of the knowledge base required by expert systems. This can become a significant handicap when it comes to generating the set of declarative rules necessary to activate the inferential mechanisms of the fuzzy logic used. Along with this, it should be noted that the concurrent counterpoint of symbolic inference and statistical inference, although less dependent on the express formalization of knowledge, implies that predictions cannot be explained and, therefore, has no plausible chain of rationality. The combination of inferential models, even hybridized, can mean an extraordinary improvement in the accuracy of diagnostic classifiers, but it is mainly the explicability of reasoning through the formalization of existing knowledge that should be considered.

## 5. Conclusions

In this paper, a novel intelligent system to support the breast cancer diagnostic process has been presented. For this purpose, the system employs, both jointly and concurrently, symbolic inference approaches (through the deployment of expert systems) and statistical inference approaches (through a statistical classifier), by means of which it is possible to determine a percentage risk metric related to the risk of suffering from breast cancer. The novelty of this system, besides the joint use of symbolic and statistical inferential approaches, lies in the way these are combined. This is achieved by means of an empirical expression that accommodates the nature of the data, as well as the possible and subsequent correction of the risk indicator using a weighting according to the BI-RADS level assigned, which allows the opinion of the medical team to be taken into account in the recommendation generated by the system.

The intelligent system was implemented in a software tool, developed based on data from the School of Medicine and Public Health of the University of Wisconsin-Madison, demonstrating its usefulness through a practical case, highlighting its simplicity and potential for further application once it is validated. In this regard, the system is currently undergoing adaptation and maturation phases in order to validated in the near future in clinical settings in order to establish its validity and reliability.

## Figures and Tables

**Table 1:** Summary of the data set.

Patients	130
Number of biopsied patients	130
Confirmed cancer cases	21
Healthy individuals (controls)	109
Average age	55.2
Number of criteria	13 Mass (shape, margins, density), calcifications (type, shape, distribution), asymmetries (type), distortion (type), breast tissue density, BI-RADS category, age, personal history and family history
Nature of the criteria	Quantitative and qualitative

**Table 2:** Summary of the patients’ general data.

Data	Data Type	Commentary
Age	Numeric	-
Patient with cancer history	Categorical	Yes, no and N/A.
Patient with family history of cancer	Categorical	None, minor, major and N/A.

**Table 3:** Summary of mammogram findings.

Subgroup	Data	Type of Data	Commentary
Mass	Shape	Categorical	None, oval, round, lobulated and irregular.
	Margins	Categorical	None, circumscribed, obscured, micro-lobulated, indistinct and spiculated.
	Density	Categorical	None, equal density, low density and high density.
Calcifications	Type	Categorical	None, primary and associated.
	Shape	Categorical	None, skin, vascular, coarse or “popcorn-like”, large rod-like, round, rim, dystrophic, milk of calcium, suture, amorphous, coarse heterogeneous, fine pleomorphic, fine linear or fine linear branching.
	Distribution	Categorical	None, diffuse, regional, grouped, linear and segmental.
Asymmetries and distortions	Type of asymmetry	Categorical	None, missing, focal and developing.
	Type of distortion	Categorical	None, primary and associated.
Breast tissue density	-	Categorical	Missing, fatty, scattered areas of fibro glandular, heterogeneously dense and extremely dense.
BI-RADS category	-	Categorical	0, 1, 2, 3, 4.a, 4.b, 4.c, 5 and 6.

**Table 4:** Weighting factors according to the order of the BI-RADS index.

**BI-RADS**	**Weighting Factor (** $F_{p}$ **)**
1	$F_{P\;}=\frac{1}{k}+f\left(R_{G}\right)\;With\;k=-BIRADS_{level}+\left(3.5\right)\;f\left(R_{G}\right)=\frac{10}{R_{G}+10}$
2	
3	$F_{c}=\frac{100}{R_{G}};\;F_{P\;}=t+f\left(R_{G}\right);\;F_{P\;}=\left\{\begin{array}{c} t+f\left(R_{G}\right)\;if\;F_{C}>F_{P}\\ \;F_{C}\;\;if\;F_{C}\leq F_{P} \end{array}\right.$ $With\;t=BIRADS_{level}-\left(1.5\right)\;f\left(R_{G}\right)=\frac{10}{R_{G}+10}$
4A	
4B	
4C	
5	$F_{P\;}=\frac{100}{R_{G}}$
6	

**Table 5:** List of MATLAB^®^ Toolboxes used in this work.

Toolbox	Commentary
App Designer [35]	Used for the design and development of the graphical user interface of the software artifact.
Fuzzy Logic Toolbox [36]	Used for the implementation of inference engines based on fuzzy logic.
Classification Learner [37]	Used for the training of classification machine learning algorithms. Allows massive and simultaneous testing of a wide variety of algorithms, making it easier for the user to select the best alternative.

**Table 6:** Summary of the antecedents and consequents of each expert system.

**Expert System 1—Masses**	
**Antecedents**	**Consequents [0, 100]**
Present/absent Shape Margins Density	R_1_
**Expert System 2—Calcifications**	
**Antecedents**	**Consequents [0, 100]**
Present/absent Type Shape Distribution	R_2_
**Expert System 3—Architectural Distortion and Asymmetries**	
**Antecedents**	**Consequents [0, 100]**
Asymmetry present/absent Type of asymmetry Distortion of the architecture present/absent Type of distortion	R_3_

**Table 7:** General configuration of expert systems’ inference engine.

Inference Engine Component	Type
Fuzzy structure	Mamdani-type
Defuzzification method	Centroid [39]
Implication method	MIN
Aggregation method	MAX

**Table 8:** System states and threshold levels.

State	Threshold
Healthy case	*Corrected Global Risk* < 40
Dubious case	40 ≤ *Corrected Global Risk* < 60
Potential case	*Corrected Global Risk* ≥ 60

**Table 9:** Results.

	Global Risk	Corrected Global Risk
Sensitivity	90.5%	100%
False negative rate	9.52%	0%
Specificity	89.81%	60.19%
False positive rate	10.19%	39.81%
Mcc	0.7	0.44
AUC	0.91	0.78
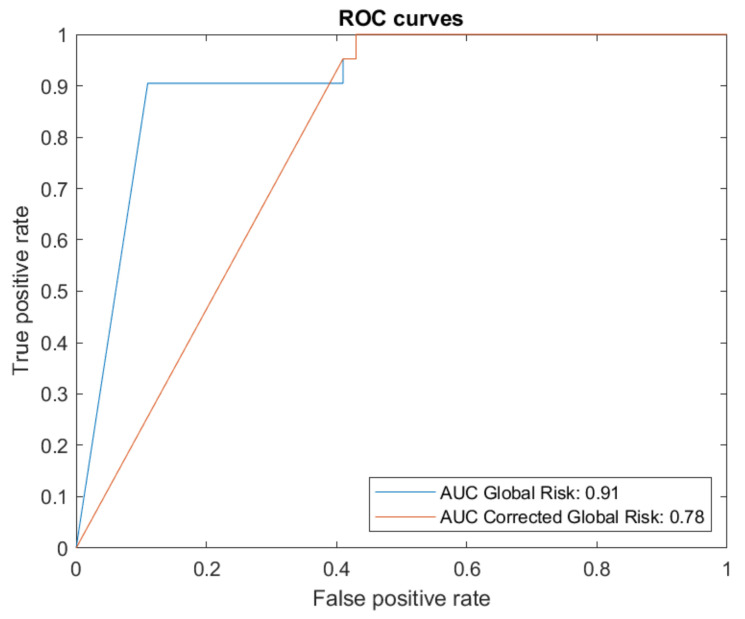		

**Table 10:** Patient’s data to be analyzed.

**Mammogram**		
**Type of finding**	**Characteristic**	**Value**
Mass	Present/absent	Present
	Shape	Irregular
	Margins	Spiculated
	Density	Homogeneous
Calcifications	Present/absent	Present
	Primary/associated	Associated
	Shape	Coarse heterogeneous
	Distribution	Grouped
Asymmetry	Present/absent	Absent
	Type	-
Architectural Distortion	Present/absent	Absent
	Primary/associated	-
Breast density	-	Heterogeneously dense
BI-RADS category	-	4B
**Other data**		
**Data**		**Value**
Age		65
Patient history		No
Family history		No

## Data Availability

The data presented in this study are available on request.
